# Supplementary material for: Synthesis of SAPO-34 Nanoplates with High Si/Al Ratio and Improved Acid Site Density
Source: Nanomaterials (Basel). 2021 Nov 25;11(12):3198. doi: 10.3390/nano11123198 (PMC8703864; doi:10.3390/nano11123198)
Supplement: Supplementary file 1 [file nanomaterials-11-03198-s001.zip › nanomaterials-1454611-supplementary.pdf]

Supplementary data

Synthesis of SAPO-34 Nanoplates with High Si/Al ration and Improved Acid Site Density

Table S1. EDX compositional analysis of the synthesized SAPO-34 particles.

	`EDX (atomic %)		
	Al	P	Si
**S1**	44.36	31.16	24.48
**S2**	48.35	46.08	5.57
**S3**	45.35	44.30	10.35
**S4**	45.06	42.01	12.93
**S5**	46.18	40.48	13.34
**S6**	47.11	38.84	14.05
**S7**	46.65	38.76	14.59
**S8**	46.72	37.03	16.25
**S9**	46.23	36.99	16.78
**S10**	44.32	39.41	16.27
**S11**	47.33	41.51	11.16
**S12**	46.68	40.81	12.54
**S13**	48.52	38.86	12.63
**S14**	48.38	40.33	11.29
**S15**	48.17	40.78	11.05
**S16**	48.15	40.79	11.06
**S17**	47.38	38.73	13.89

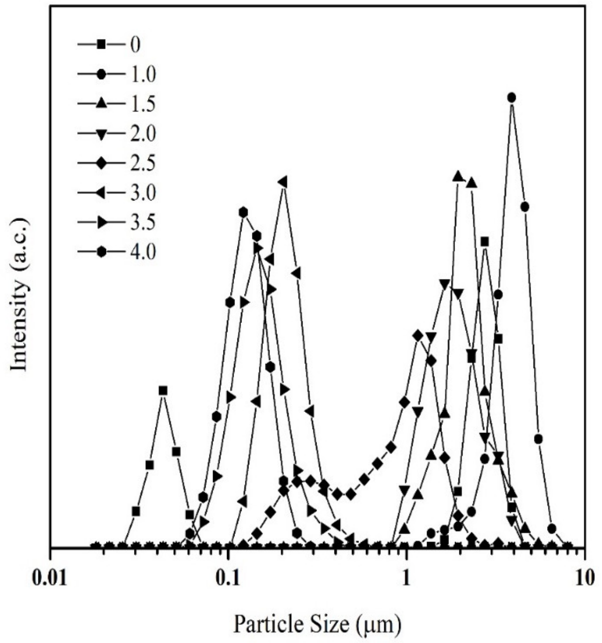

Figure S1. DLS for the SAPO-34 particles with varied template concentration.

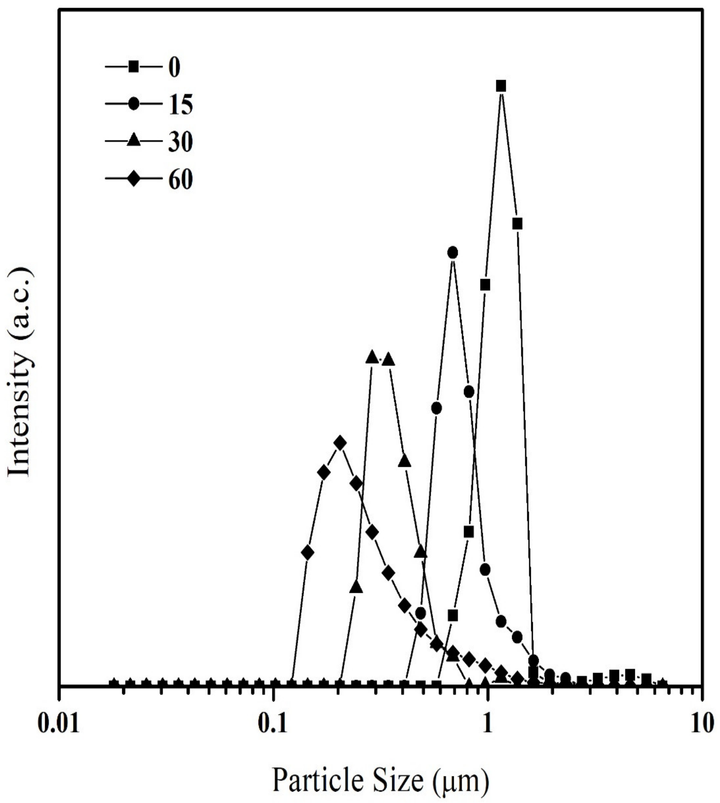

Figure S2. DLS data for the synthesized particles with variation of time for phosphoric acid addition.

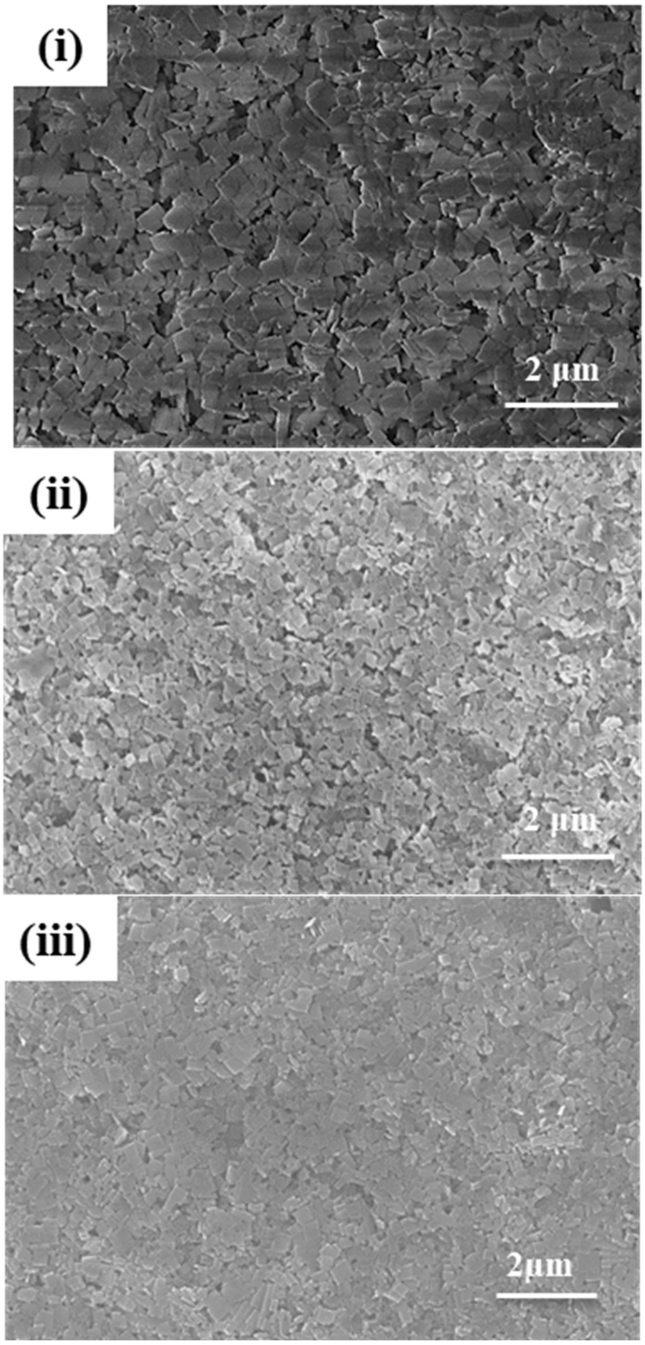

Figure S3. (a) SEM images for the samples with variation of Si concentration z = (i) 0.4, (ii) 0.5, and (iii) 0.6. Composition: $1Al_2O_3$: 4TEAOH: $2P_2O_5$: $zSiO_2$: $100H_2O$.

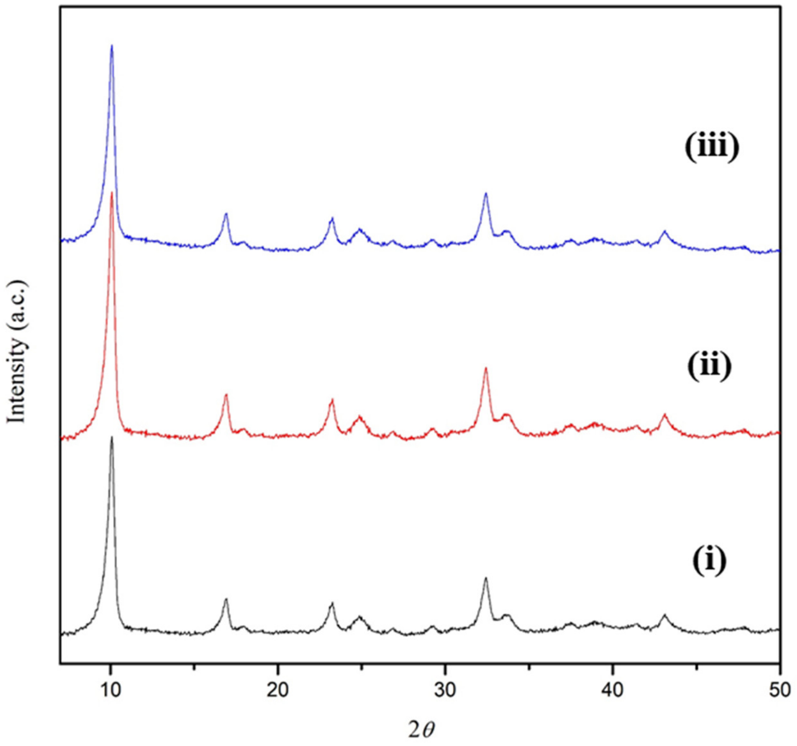

Figure S3. (b) XRD images for the samples with variation of Si concentration z = (i) 0.4, (ii) 0.5, and (iii) 0.6.

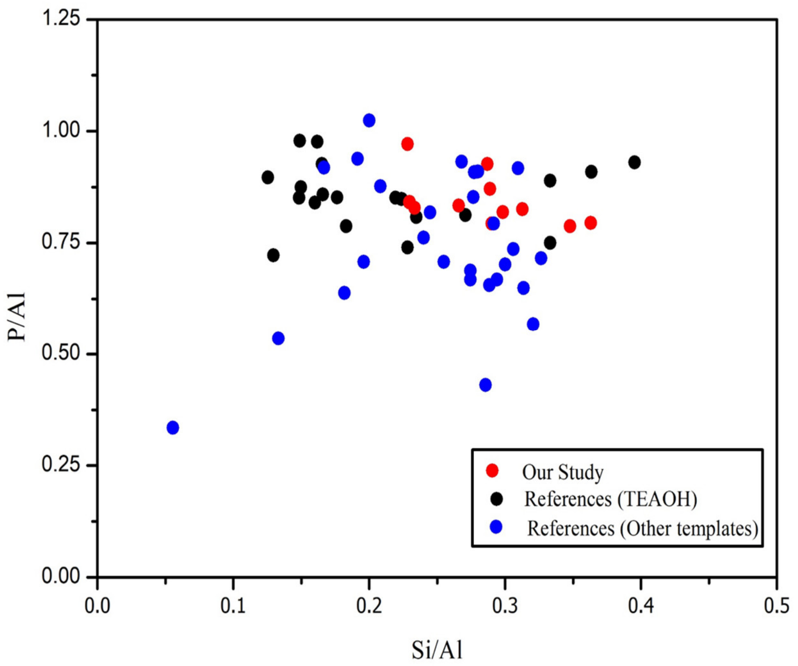

Figure S4. A comparison of the composition of SAPO-34 particles from literature for their Si/Al and P/Al ratio.

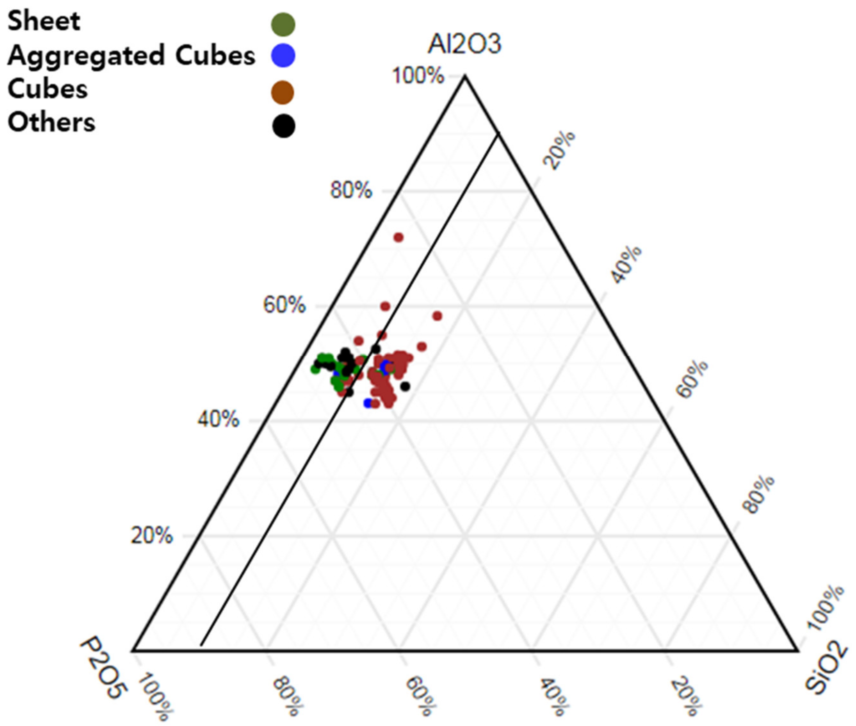

Figure S5. The morphological distribution for various composition SAPO-34 particles in the literature.

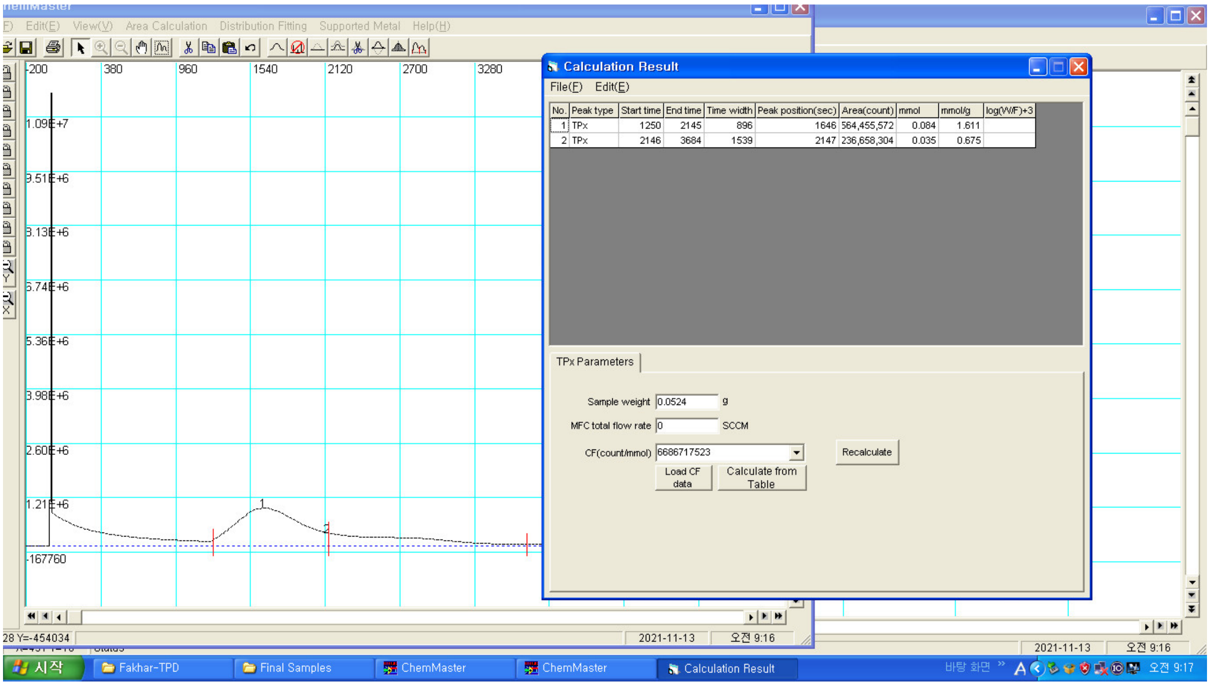

Figure 6. The TPD data was analyzed using ChemMaster software, where the peaks were evaluated for weak and strong acid sites.
